# Hyper-activation of Aurora kinase a-polo-like kinase 1-FOXM1 axis promotes chronic myeloid leukemia resistance to tyrosine kinase inhibitors

**DOI:** 10.1186/s13046-019-1197-9

**Published:** 2019-05-23

**Authors:** M. Mancini, S. De Santis, C. Monaldi, L. Bavaro, M. Martelli, F. Castagnetti, G. Gugliotta, G. Rosti, M. A. Santucci, G. Martinelli, M. Cavo, S. Soverini

**Affiliations:** 1 Dipartimento di Medicina Specialistica, Diagnostica e Sperimentale – DIMES - Istituto di Ematologia “L. e A. Seràgnoli”, University of Bologna, Medical School, via Massarenti, 9, 40138 Bologna, Italy; 2 Istituto Scientifico Romagnolo per lo Studio e la Cura dei Tumori (IRST) Srl Istituto di Ricovero e Cura a Carattere Scientifico (IRCCS), via Piero Maroncelli 40, 47014 Meldola (FC), Italy

**Keywords:** Chronic myeloid leukemia, Drug resistance, Aurora kinase a, Polo-like kinase 1, FOXM1, β-Catenin

## Abstract

**Background:**

Chronic myeloid leukemia (CML) is a myeloproliferative disease caused by the constitutive tyrosine kinase (TK) activity of the BCR-ABL1 fusion protein. Accordingly, TK inhibitors have drastically changed the disease prognosis. However, persistence of the transformed hematopoiesis even in patients who achieved a complete response to TK inhibitors and the disease relapse upon therapy discontinuation represent a major obstacle to CML cure.

**Methods:**

Thiostrepton, Danusertib and Volasertib were used to investigate the effects of FOXM1, AKA and Plk1 inhibition in K562-S and K562-R cells. Apoptotic cell death was quantified by annexin V/propidium iodide staining and flow cytometry. Quantitative reverse transcription (RT)-PCR was used to assess BCR-ABL1, FOXM1, PLK1 and AURKA expression. Protein expression and activation was assessed by Western Blotting (WB). Clonogenic assay were performed to confirm K562-R resistance to Imatinib and to evaluate cells sensitivity to the different drugs.

**Results:**

Here we proved that BCR-ABL1 TK-dependent hyper-activation of Aurora kinase A (AURKA)-Polo-like kinase 1 (PLK1)-FOXM1 axis is associated with the outcome of Imatinib (IM) resistance in an experimental model (K562 cell line) and bone marrow hematopoietic cells. Notably, such a biomolecular trait was detected in the putative leukemic stem cell (LSC) compartment characterized by a CD34+ phenotype. Constitutive phosphorylation of FOXM1 associated with BCR-ABL1 TK lets FOXM1 binding with β-catenin enables β-catenin nuclear import and recruitment to T cell factor/lymphoid enhancer-binding factor (TCF/LEF) transcription complex, hence supporting leukemic cell proliferation and survival. Lastly, the inhibition of single components of AURKA-PLK1-FOXM1 axis in response to specific drugs raises the expression of growth factor/DNA damage-inducible gene a (GADD45a), a strong inhibitor of AURKA and, as so, a critical component whose induction may mediate the eradication of leukemic clone.

**Conclusions:**

Our conclusion is that AURKA, PLK1 and FOXM1 inhibition may be considered as a promising therapeutic approach to cure CML.

**Electronic supplementary material:**

The online version of this article (10.1186/s13046-019-1197-9) contains supplementary material, which is available to authorized users.

## Background

A single genetic lesion, the t (9,22) reciprocal translocation which generates the BCR-ABL1 rearranged gene, is the causative event of CML, driving clonal expansion of leukemic hematopoiesis via the constitutive activation of BCR-ABL1 TK [1]. Targeted therapy with TK inhibitors (imatinib [IM], nilotinib or dasatinib, has drastically changed the disease prognosis [2]. However, development of drug resistance and persistence of minimal residual disease despite continued therapy suggest that the coexistence of additional events support the advantage of transformed clone over the normal counterpart [3]. In particular, minimal residual disease persistence and molecular relapses that may occur following treatment discontinuation suggest that BCR-ABL1-independent signals sustain the maintenance of a pool of transformed cells potentially able to drive the disease progression towards its fatal outcome, the blast crisis. Indeed, BCR-ABL1+ LSC are intrinsically resistant to TK inhibitors and incompletely eradicated by therapy in most patients [4, 5]. CML LSCs evade from TK inhibitor-induced death through multiple pathways, including micro-environmental niche regulatory molecules, Hedgehog (Hh), WNT, polycomb gene BMI1 and Notch [6]. Genomic instability associated with the persistence of BCR-ABL1 fusion gene may have a central role in the selection of further genetic aberrations in leukemic hematopoiesis [7]. Therefore, there is currently a great interest in the comprehension of signals promoting LSC proliferation and maintenance, in an attempt to develop eradicating strategies for CML patients.

Our recent work has proven the hyper-activation of PLK1 serine/threonine kinase and FOXM1 transcription factor in the putative stem cell compartment of CML, identified by CD34+ phenotype, and their role in BCR-ABL1+ LSC persistence under TK inhibitor therapy [8]. We extended our investigation to Aurora kinase A (AURKA), the upstream signal of PLK1-FOXM1 axis, whose inhibition has been used for clinical purposes to overcome CML drug resistance [9]. Multiple events drive AURKA-PLK1-FOXM1 axis hyper-activation associated with BCR-ABL1 TK: the constitutive activation of AKT, which activates AURKA, enhanced production of reactive oxygen species (ROS) and inactivation of protein phosphatase 2A (PP2A), promoting PLK1 activation, MELK phosphorylation by PLK1 – which drives FOXM1 activation – and reduced SUMOylation – which enhances protein stability. Moreover, feed-back pathways including activated MAPK/ERK and recruitment and transactivation of FOXM1 target genes concur to keep AURKA hyper-activated [10–17]. The enhanced phosphorylation of histone H3 at Ser^10^, whose recruitment at target promoters may be involved in transcription of genes affecting the leukemic phenotype, is a further component of AURKA-driven proliferative and survival advantage of BCR-ABL1+ hematopoiesis (see Graphical abstract) [18]. Moreover, AURKA-PLK1-FOXM1 axis interaction with β-catenin, a key effector of WNT pathway in normal and leukemic stem cells, supports its participation in the persistence of BCR-ABL1+ LSC, in spite of the inhibition of fusion protein enzymatic activity in response to TK inhibitors [19–22]. The dual role of AURKA-PLK1-FOXM1 axis in proliferation and DNA damage repair makes its components good targets for leukemia eradication.

## Materials and methods

### Study population

Ten CML patients in chronic phase of the disease and three CML patients in chronic phase that are resistant to TKI without mutations in BCR-ABL1, were included in our study. All of them exhibited the BCR-ABL1 rearranged gene coding for p210-kDa fusion protein. Ten CML patients in chronic phase achieved a complete hematological response (CHR) at the 3rd month of therapy with TK inhibitors. Eight patients achieved a major molecular response (MMR: 3 log reduction of BCR-ABL1 transcript levels compared to diagnosis) within the 1st year of therapy and the follow-up of remaining 3 patients was not evaluable (NE) because of the too short interval since treatment starting. The mononuclear cell fraction (MNC) from bone marrow samples of patients and peripheral blood apheresis products of healthy donors (HD) were obtained by means of Ficoll-Hypaque gradient. Equal amounts of RNA and proteins from peripheral blood samples of 8 HDs were pooled to avoid individual differences in transcript and protein expression. CD34+ cells were isolated from bone marrow samples of CML patients at diagnosis and apheresis products of 8 HDs by immunomagnetic separation (mini-MACS from Miltenyi Biotec). Briefly, MNC (1–5 × 10^8^/mL) were incubated at 4 °C for 15′ with magnetic microbeads coated with anti-CD34 antibody (Miltenyi Biotec). CD34+ cells were flown through a separation column in a magnetic field. Cell purity was confirmed using flow cytometric analysis of anti-CD34-FITC antibody (BD Biosciences); it was > 90% in all cases (data not shown).

Investigators performing the experiments were blinded with respect to the WHO subtype, clinical features and outcome of the patients. Sample- and data collection were approved by the Institutional Review Boards of the S. Orsola-Malpighi Hospital (protocol 112/2014/U/Tess).

### Cells and treatments

IM sensitive and IM-resistant K562 BCR-ABL1+ (K562-S, K562-R), 32Dp210-S and -R, BaF3-S and -R cell lines were maintained in RPMI 1640 medium (Lonza) supplemented with 10% fetal calf serum (FCS, Gibco), 1% L-Glutamine and antibiotics in 5% CO2 and fully humidified atmosphere at 37 °C [9]. To inhibit BCR-ABL1 TK, PLK1, AURKA and FOXM1 Imatinib (IM: 1 μM), Volasertib (1 μM), Thiostrepton (1 μM) and Danusertib (1 μM) were additioned to culture media for 24 h. Apoptotic cell death was measured by the uptake of fluorescinated Annexin V and propidium iodide (PI, both from Roche) using a FACsCantoII flow cytometer (Beckton Dickinson) set at 488 nm excitation and 530 nm wavelength bandpass filter for fluorescin detection or 580 nm for PI detection. Results obtained were analyzed using a dedicated software (DIVA software, Beckton Dickinson).

### Cytofluorimetric analysis of apoptosis induction

Cytofluorimetric analysis of apoptotic cell fraction was performed by measuring the uptake of Annexin V (Hoffmann-La Roche, Basel, SW) and propidium iodide (PI) (Sigma) according to published methods (41). Cell fluorescence and PI uptake were measured by mean of a FACScan flow cytometer (set at 488 nm excitation and 530 nm bandpass filter wave length for fluorescin detection or 580 nm for PI detection) and a dedicated software (both from Beckton Dickinson).

### Quantitative reverse transcription (RT)-PCR for BCR-ABL1, FOXM1, PLK1 and AURKA expression

A commercial kit (SV total RNA Isolation System, Promega) was used for total RNA extraction starting from 1 × 10^6 cells. In the K562-S and R cell lines.

BCR-ABL1, FOXM1, PLK1 and AURKA expression was assessed by quantitative RT-PCR. Total RNA (200 ng) was reverse transcribed to cDNA with the High-Capacity cDNA Reverse Transcription Kit (Thermo Fisher Scientific, Waltham, MA, USA). Assays were performed in triplicate on the ABI 7900HT system (Thermo Fisher Scientific) using pre-designed TaqMan Gene Expression Assays (Thermo Fisher Scientific) for BCR-ABL1, FOXM1, PLK1, AURKA and B2M as control gene.

### Protein analysis

Western blot (WB) and immunoprecipitation /immunoblotting (IP/IB) analyses were performed on whole cell lysates and nuclear fractions according to published methods [23, 24]. The anti-β-catenin, anti-PLK1, anti-phospho-PLK1 (Thr210), anti-AURKA, anti-phospho-AURKA (Thr288) and anti-GADD45α (D17E8) were purchased from Cell Signaling Technology. The anti-FOXM1 was purchased from Abcam. The anti-β-actin antibody used as loading control was purchased from Santa Cruz Biotechnology. Signal intensities in single blots obtained in three separate experiments were measured by means of ChemiDoc-It instrument (UVP) equipped with a dedicated software (Launch VisionWorksLS from Euroclone). The differences among signal intensities were evaluated for statistical significance using the paired Student’s t-test.

### Clonogenic assay

Drug cytotoxicity was evaluated by clonogenic assays. The reduction of colony (generated in 0.9% methylcellulose supplemented with 30% fetal calf serum) number in the presence of increasing doses of Imatinib (0.025–0.1 μM), Thiostrepton (0.025–0.1 μM), Danusertib (0.025–0.1 μM) and Volasertib (0.025–0.1 μM) was assessed after 14 days of incubation at 37 °C in a fully humidified atmosphere and 5% CO2. Thiostrepton (0.05–0.30 μM), Danusertib and Volasertib (0.025–0.100 μM) were also tested in cells from three CML patients resistant to TKI without mutations in BCR-ABL1 and one healthy donor, for comparison. Nonlinear regression analyses (GraphPad Software Inc., La Jolla, CA) were used to calculate the lethal dose (LD50) of different drugs in cell lines and in primary patient cells.

## Results

### The up-modulation of AURKA/PLK1/FOXM1 axis is a component of IM resistance in BCR-ABL1+ cell line

**Fig. 1:**
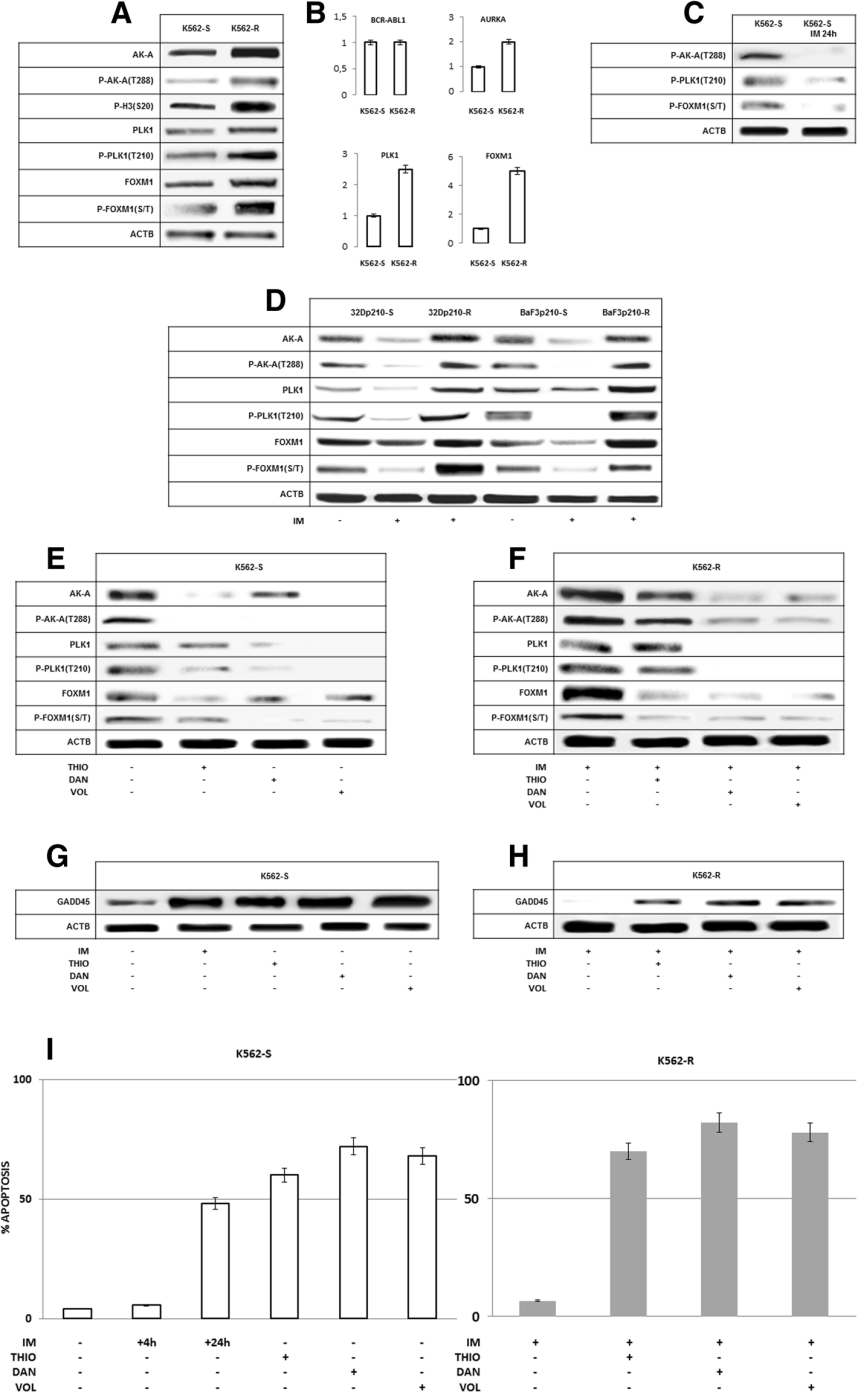
The up-modulation and hyper-activation of Aurora A/PLK1/FOXM1 axis associated with IM resistance in BCR-ABL1+ cell lines. **a**-Aurora A expression and phosphorylation at T288 are significantly increased in IM-resistant K562 cells, and result in hyper-phosphorylation of its target, the histone H3 at S20. IM resistance is associated with the over-expression and hyper-activation of downstream signals: PLK1 and FOXM1; **b**-A significant increment of Aurora A, PLK1 and FOXM1 transcripts (*p* < 0.05 or less) supports that the over-expression of these three signals associated with drug resistance is partly contingent upon transcriptional events; **c**- Aurora A, PLK1 and FOXM1 phosphorylation is significantly reduced in response to IM, supporting their dependence from BCR-ABL1 TK; **d**- Aurora A expression and phosphorylation at T288 are significantly increased in IM-resistant 32Dp210 and BaF3p210 cells. IM resistance is associated with the over-expression and hyper-activation of downstream signals: PLK1 and FOXM1. A reduction of AURORA A, PLK1 and FOXM1 expression and activation was evident in IM-sensitive and IM-resistant cell lines in response to pan-AK inhibitor Danusertib (Dan), PLK1 inhibitor Volasertib (Vol) and FOXM1 inhibitor Thiostrepton (Thio); **e**,**f**-A likewise reduction of AURORA A, PLK1 and FOXM1 expression and activating phosphorylation was apparent in IM-sensitive and IM-resistant K562 in response to pan-AK inhibitor Danusertib (Dan), PLK1 inhibitor Volasertib (Vol) and FOXM1 inhibitor Thiostrepton (Thio); **g,h**-AURORA A inhibitory signal GADD45a is significantly raised in response to IM in IM-sensitive K562 cell line and to Dan, Vol and Thio both in IM-sensitive and IM-resistant K562 cells **I**-Apoptotic death induction in response to above mentioned drugs of IM-sensitive and IM-resistant K562 cells

To investigate if IM resistance in a BCR-ABL1+ cell context is associated with the over-expression and hyper-activation of AURKA, PLK1 and FOXM1 axis, IM resistance was induced in the K562, 32D p210 and BaF3 p210 cell lines by exposure to progressively increasing doses of IM (from 0.1 up to 8 μM). IM resistance was validated by dose-response curves of cells grown in the presence of 8 μM IM since the end of the 2nd month of selection, showing significant differences in LD_50_ of IM-sensitive K562 cells (K562-S) as against IM–resistant K562 cells (K562-R; 0.0293 μM vs. 0.1760 μM, respectively) (Additional file 1: Figure S1). AURORA A expression and activating phosphorylation were significantly increased in all the resistant cell lines as compared to sensitive ones (Fig. 1a, d). As expected, AURKA hyper-phosphorylation observed in IM resistant cells was associated with a significant increase in the phosphorylation of its target H3 at Ser^20^, and of PLK1 and FOXM1 expression and phosphorylation (Fig. 1a, d). AURKA, PLK1 and FOXM1 up-modulation associated with IM resistance is partly contingent upon transcriptional events. Using a real time PCR assays we found a significant increase of AURKA, PLK1 and FOXM1 transcripts in K562-R compared to IM-sensitive cells, with no differences in BCR-ABL1 transcript levels (Fig. 1b). The enhanced AURKA transcription rate is most likely driven by epigenetic mechanisms, namely the histone H3 de-phosphorylation at Ser10 independent from BCR-ABL1 TK activity illustrated in our previous study [18].

In the K562-S, 32Dp210-S and BaF3p210-S cell lines, BCR-ABL1 TK inhibition in response to 24 h exposure to 1 μM IM induced a significant reduction of AURKA, PLK1 and FOXM1 phosphorylation, indicating that AURKA, PLK1 and FOXM1 are components of a unique loop activated by the BCR-ABL1 TK (Fig. 1c, d). Further experiments showed a significant reduction and inactivation of AURKA, PLK1 and FOXM1 in response to Thiostrepton (1 μM) a FOXM1 inhibitor, Volasertib (1 μM) a PLK1 inhibitor and Danusertib (1 μM) a pan-AK inhibitor, respectively, both in the K562-S and in the K562-R cell line (Fig. 1e and f).

The stress sensor gene GADD 45a, which physically associates with AURKA and strongly inhibits its activity, is a putative intermediary of AURKA-PLK1-FOXM1 down-regulation in response to IM and other mentioned drugs [25]. Indeed, GADD45a expression was significantly increased in response to IM, Thiostrepton, Volasertib and Danusertib in K562-S and to Thiostrepton, Volasertib and Danusertib in K562-R cells (Fig. 1g and h).

Apoptotic cell death is the expected outcome of BCR-ABL1, FOXM1, PLK1 and AURKA inactivation in response to specific inhibitors. Accordingly, the number of apoptotic cells increased up to 50% by drug treatment in K562-S and -R cell lines (Fig. 1i).

### FOXM1 de-phosphorylation in response to the inhibition of AURKA-PLK1-FOXM1 axis promotes β-catenin release preventing its nuclear import and transcription

**Fig. 2:**
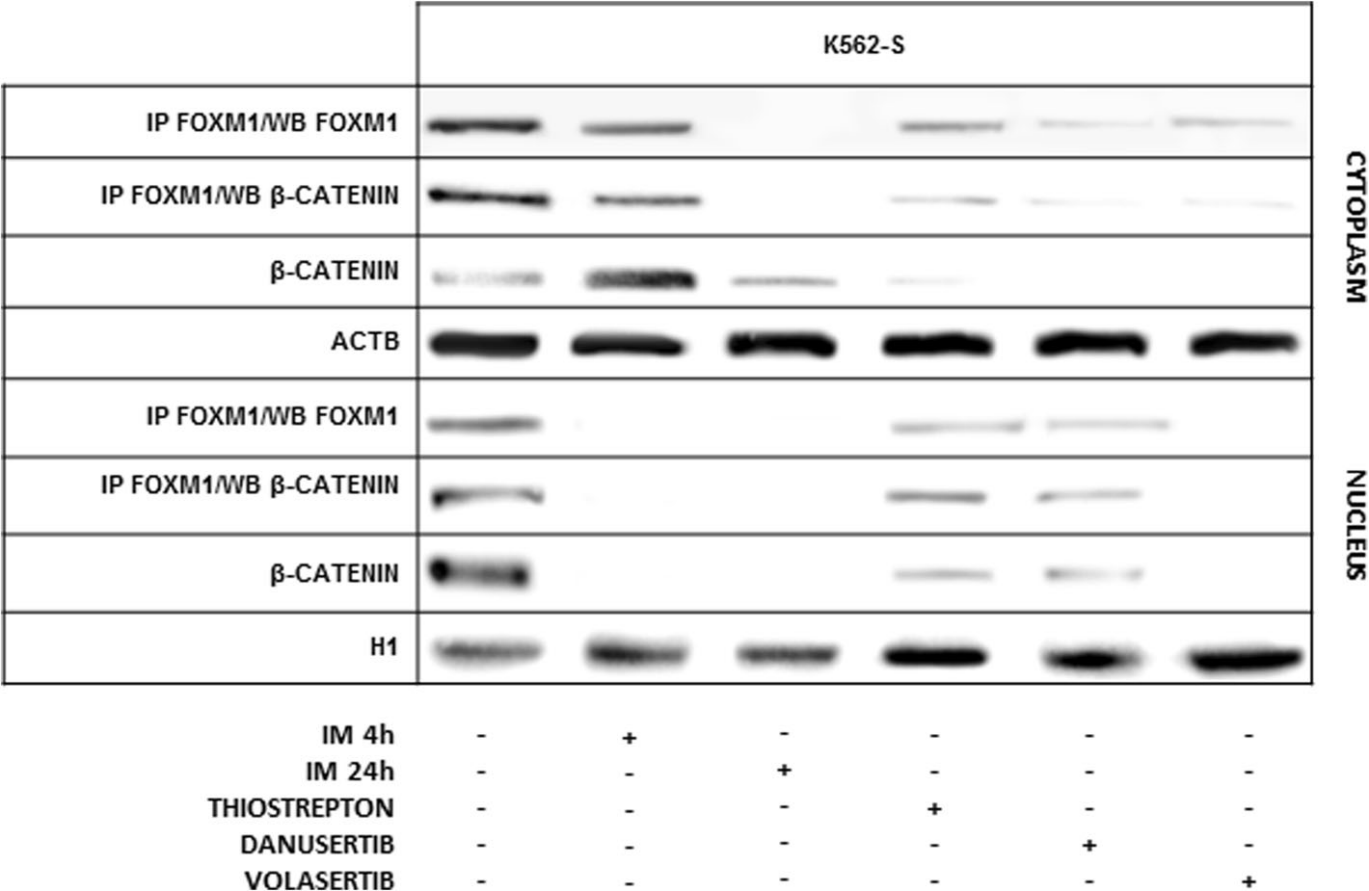
FOXM1 de-phosphorylation in response to Aurora A/PLK1/FOXM1 inhibition drives β catenin release and nuclear import. FOXM1 de-phosphorylation in response to IM, Vol, Dan and Thio prevents β catenin binding in the cytoplasmic and nuclear compartment of IM-sensitive K562 cells (*p* < 0.01 or less). β catenin decay following its release from de-phosphorylated FOXM1 is likely driven by nuclear export and cytoplasmic degradation

FOXM1 impact on cell proliferation is due to its interaction with β-catenin promoting, in turn, β-catenin nuclear import and recruitment to TCF/LEF transcription activation complex to trans-activate target genes [21]. Accordingly, FOXM1 inactivating de-phosphorylation following BCR-ABL1 TK, FOXM1, PLK1 and AURKA inhibition, affected β-catenin binding with FOXM1 in the cytoplasmic and nuclear compartments (Fig. 2). In the cytoplasmic compartment of K562-S cells the dissolution of FOXM1/β-catenin complex was apparent since the 4th hour of IM exposure and was fully achieved by the 24th hour, while β-catenin release from FOXM1 in the nuclear compartment was almost completed at the 4th h (Fig. 2). Previous studies proved that β-catenin nuclear export following de-phosphorylation and/or release from molecules involved in sub-cellular traffic addresses β-catenin towards proteasomal degradation, hence promoting its reduction in the cytoplasmic compartment [26, 27]. Finally the same effects on β-catenin-FOXM1 complex and β-catenin stability in the cytoplasmatic compartment were observed after Thiostrepton, Volasertib and Danusertib treatment both in K562-S and -R cell lines (Fig. 2 and Additional file 2: Figure S2).

### AURORA A-PLK1-FOXM1 axis up-modulation in clonal hematopoietic progenitors of IM-resistant versus IM-sensitive patients

**Fig. 3:**
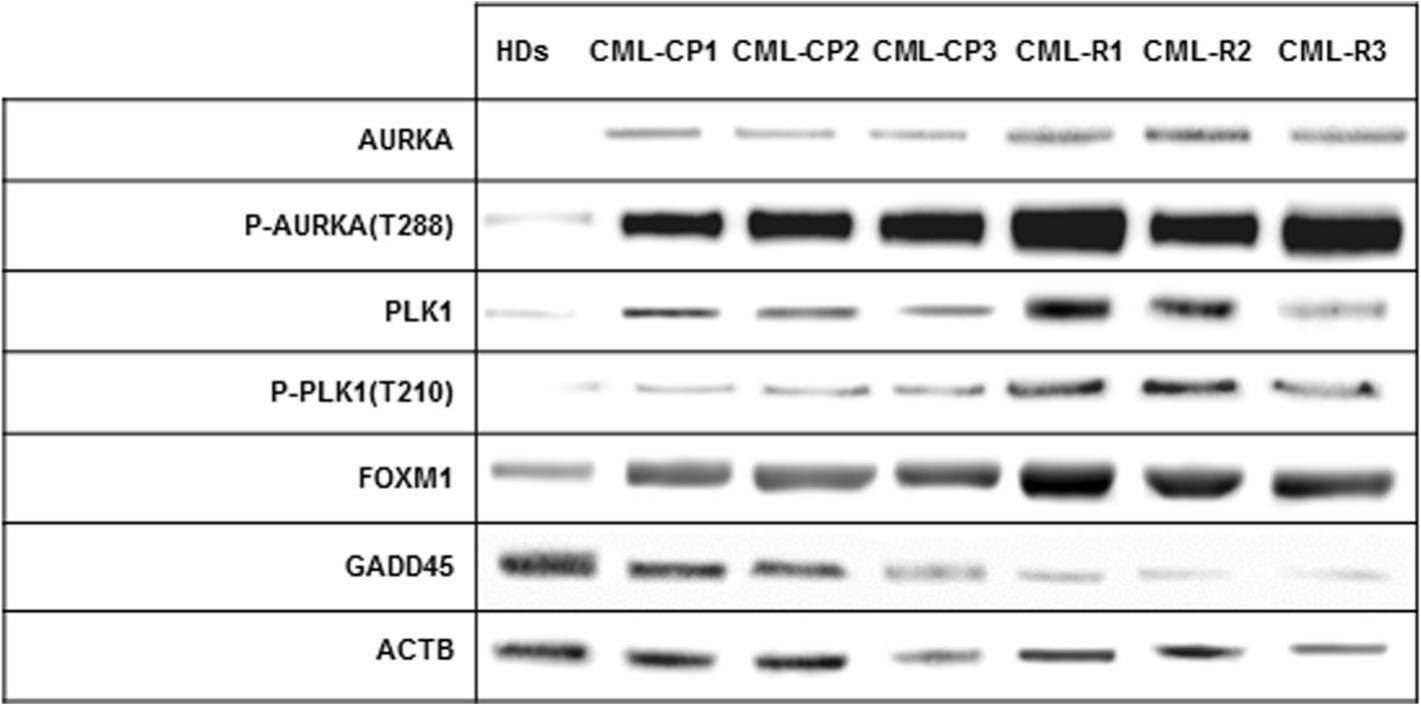
Aurora A/PLK1/FOXM1 axis is up-modulated in clonal hematopoiesis of IM-resistant CML patients. Aurora A/PLK1/FOXM1 expression and activating phosphorylation were analyzed in MNCs from bone marrow samples of CML patients at diagnosis and IM-resistant CML patients

We next confirmed in an ex vivo model the results obtained in K562 cell lines. To this purpose, MNC isolated from bone marrow samples of CML patients at diagnosis were compared with MNC from bone marrow of CML patients who developed resistance to TK inhibitors for AURKA, PLK1 and FOXM1 expression levels and phosphorylation status. All samples exhibited similar levels of BCR-ABL1 protein and transcript (data not shown). In first instance, we found slightly significant differences in AURKA expression in the MNC fraction from CML patient who responded to TK inhibitors compared to a pool of peripheral blood apheresis from healthy donors (*p* < 005). Such a difference was significantly greater in MNCs from drug-resistant patients (*p* < 0.01) (Fig. 3). AURKA activating phosphorylation at Thr288 exhibited a significant increase in the MNCs from CML patients at diagnosis compared to the normal control pool, further significantly increased in the MNCs from drug resistant patients (*p* < 0.01 or less) (Fig. 3). Moreover, we confirmed the over-expression and hyper-phosphorylation of PLK1 and FOXM1 in the MNCs from CML patients at diagnosis compared to the healthy donor pool (Fig. 3) [8]. The further and significant increase of PLK1 and FOXM1 expression and activating phosphorylation in MNCs from IM-resistant CML patients reinforces the hypothesis of participation of those two signals in the development of drug resistance advanced by results obtained in K562-R cells (Fig. 3). Notably, in this cell context AURKA activation was negatively correlated with the expression of GADD 45a, supporting the interaction of two signaling pathways involved in cell cycle regulation and genomic stability published in a previous paper (Fig. 3) [28].

### AURKA-PLK1-FOXM1 axis has a central role in the resistance to TK inhibitors of CML LSC

**Fig. 4:**
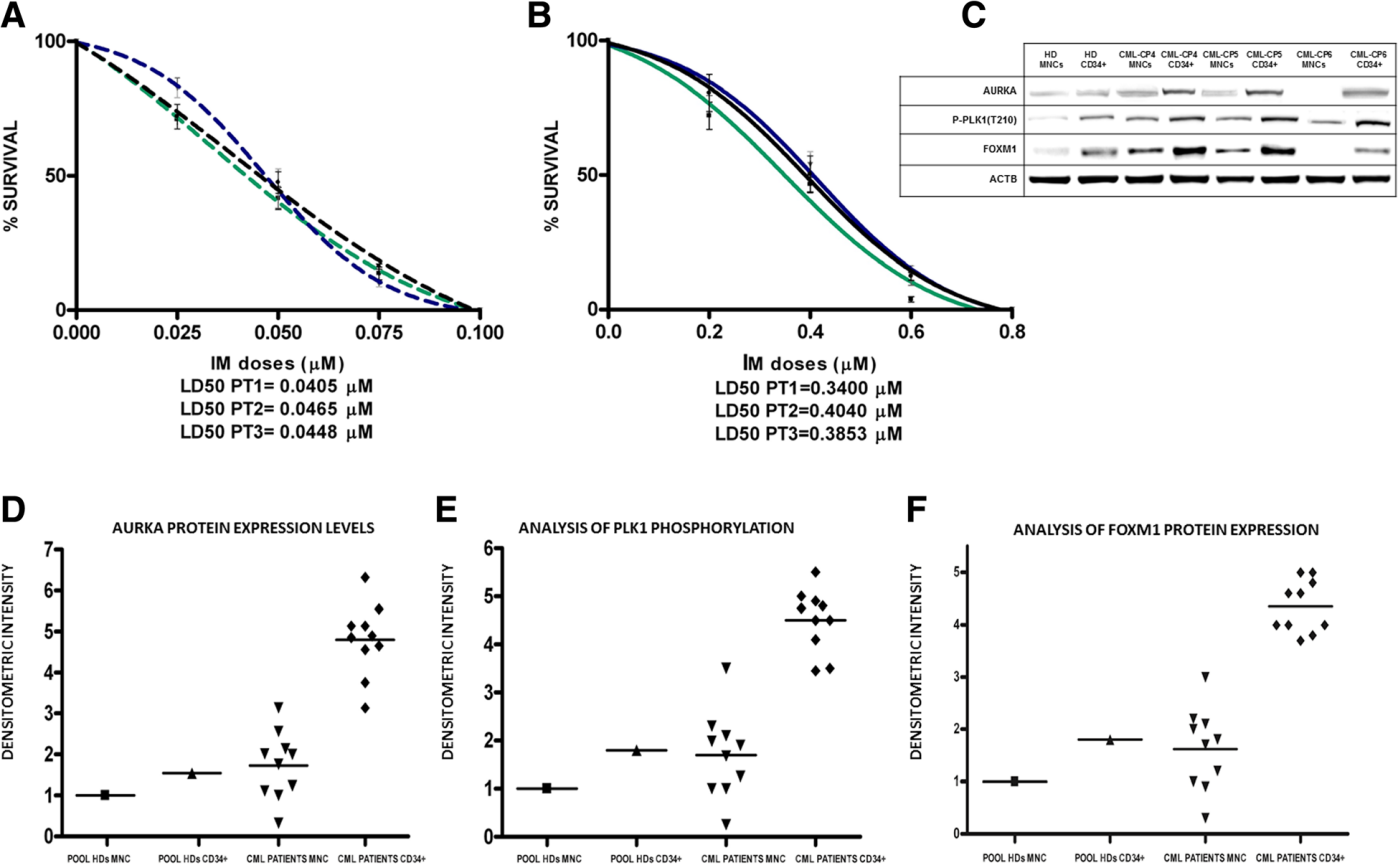
Aurora A/PLK1/FOXM1 axis is hyper-activated in CD34+ compartment from CML patients at diagnosis compared to a pool of 8 HD. **a**-MNCs from three CML patients at diagnosis were sensitive to IM administration with LD_50_ ranging from 0.0405 to 0.0465 μM; **b**- Ph1+/CD34+ cells separated from the same three patients exhibited a relative resistance to IM, with LD_50_ ranging from 0.3400 to 0.4040 μM; **c-d-e-f**-Aurora A overexpression, PLK-1 hyper-activitation and FOXM1 overexpression is restricted to CD34+ compartment of ten CML patients. Notably, Aurora A and FOXM1 protein expression and PLK1 hyper-phosphorylation were significantly higher in CD34+ cells compared to MCF of HD (panels **c**, **d**, **e** and **f**), suggesting Aurora A, PLK1-FOXM1 axis is a stemness component in the hematopoietic tissue. In panels **d**, **e** and **f** the values of protein expression and phosphorylation in MNCs and CD34+ cells of individual CML patients relative to the HD pool were obtained by comparison of band densitometry analysis (see Materials and Methods section for details)

*The last point of our investigation was aimed to investigate whether AURKA-PLK1-FOXM1 axis participates in the resistance to TK inhibitors of CML LSC. To this purpose, we choose to compare AURKA-PLK1-FOXM1 expression and activating phosphorylation in CD34+ cells purified from bone marrow samples of CML patients at diagnosis and peripheral blood of healthy persons pooled to avoid individual differences* [24]*. Indeed, CML LSC supposedly reside within the CD34+/CD38−/Lin- fraction, which is the same compartment where also normal HSC are found. More recently CD26 has been identified as a better marker of the leukemic clone* [28]*. Preliminary experiments confirmed the extreme paucity of CD34+/CD38−/Lin−/CD26+ compartment (range: 0.007–087/*μL*), absolutely insufficient to perform any further analysis (data not shown)* [29]*. Moreover, such a compartment is heavily contaminated by normal, Ph1- hematopoietic stem cells* [30]*. On the other hand, the CD34+ compartment is almost completely composed of leukemic Ph1+ cells, still owning a sufficient degree of stemness to engraft immunodeficient mouse strain animals and generate aggregates (colonies) made of pluripotent cell types in semi-solid culture media* [30]*. The Ph1 chromosome was seen in > 93% of CD34+ cells (data not shown). Moreover, Ph1+/CD34+ cells exhibited a relative resistance to IM, with LD*_*50*_ *ranging from 0.3400 to 0.4040* μM*, compared to the LD50 ranging from 0.0405 and 0.0448 μM of bone marrow MNCs (*Fig. 4a and b*). Comparing the signal intensities of single patient blots relative to the signal intensity of a pool of MNC of normal donors (considered equal to 1), we found a significantly higher expression of AURKA, phosphorylated PLK1 and FOXM1 in the CD34+ compartment compared to the MNCs of the corresponding patient (*Fig. 4c, d, e, f*).*

**Fig. 5:**
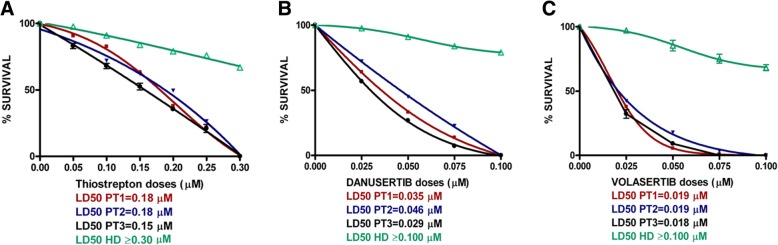
Effects of Thiostrepton, Danusertib and Volasertib on mononuclear cells from three patients resistant to TKIs without mutations on BCR-ABL1 . Reduction of clonogenic growth of cells from 3 patients with CML (blue, red and black curves) as compared to a healthy donor (HD; green curve) in the presence of increasing doses of Thiostrepton (0.05–0.30 μM), Danusertib and Volasertib (0.025–0.100 μM). Nonlinear regression analyses (GraphPad Software Inc., La Jolla, CA) were used to calculate the lethal dose (LD50) of different drugs in cell lines and in primary patient cells

*Finally, we assessed the effects of Thiostrepton, Danusertib and Volasertib on clonogenic potential in neoplastic mononuclear cells from three patients with CML resistant to TKIs without mutations in BCR-ABL1. All the approaches induced a dose-dependent reduction in colony formation (with LD50 ranging from 0.15 to 0.18 μM for Thiostrepton, from 0.029 to 0.046 μM for Danusertib and from 0.018 to 0.019 μM for Volasertib) (*Fig. 5a, b, c*). Comparison with the effects on cells from healthy donor, tested as controls, showed that the extent of growth inhibition was strictly related with neoplastic phenotype.*

## Discussion

CML progression is driven by multiple genetic and epigenetic events, including amplification of BCR-ABL1, increased activity of BCR promoter, impaired activity of PP2A, inhibition of SRC homology region 2 domain-containing phosphatase-1 (SHP1) and hyper-methylation of tumor suppressor genes involved in the control of proliferation and survival [31]. Such mechanisms provide complementary routes to proliferation and survival of the transformed clone and, more importantly, promote the persistence of a Ph1+ quiescent LSC pool, which may eventually drive disease relapse and drug resistance. Accordingly, LSC selective inhibition combined with standard TK inhibitor therapy is regarded as the most promising approach to CML cure. The success of such integrated strategy is contingent upon the identification of signaling hubs which control LSC proliferation and survival and may let the selective targeting of leukemic hematopoiesis.

We have recently demonstrated the hyper-activation of the PLK1-FOXM1 axis associated with the BCR-ABL1 TK activity in the putative LSC compartment identified by a CD34+ phenotype [8]. Results presented here prove that AURKA, a component of the mitosis regulatory network, interacts with PLK1 and FOXM1 to promote IM resistance. AURKA, a serine/threonine kinase required for centrosome function and spindle assembly, is regulated by transcriptional and post-transcriptional events. Its activation at the G2/M transition is promoted by auto-phosphorylation by glycogen synthase kinase 3 beta (GSK-3β) and cyclin-dependent kinase 1 (CDK1). At mitotic exit, it undergoes destruction by the anaphase promoting complex /cyclosome (APC/C)-cadherin 1 (Cdh1) following inactivation by protein phosphatase 1γ (PP1γ) and PP2A de-phosphorylation [32–34]. Once activated, AURKA impacts on multiple targets involved in centrosome maturation, spindle assembly, cell cycle progression and downstream gene transcription. In particular, AURKA, in complex with its co-factor Bora, phosphorylates PLK1 at Thr210, the prerequisite for PLK1 to promote G2 checkpoint recovery and a central event for regulation of centrosome maturation, bipolar spindle formation and chromosome alignment [35, 36]. FOXM1 is a critical substrate of PLK1 involved in cell proliferation, cell cycle progression and genomic stability. Its phosphorylation by PLK1 controls the execution of FOXM1 transcriptional program required for mitotic progression and involved in chemo-resistance, DNA damage repair and MELK signaling up-regulation [37–39] (see graphical abstract).

Here we showed the over-expression and hyper-activation of all three components of AURKA-PLK1-FOXM1 axis associated with BCR-ABL1 TK and their further increment associated with drug resistance in BCR-ABL1+ cell line K562 (Fig. 1a). The results were validated in bone marrow samples of CML patients, where a significant increase of AURKA, PLK1 and FOXM1 activating phosphorylation was associated with BCR-ABL1 TK and IM resistance (Fig. 3). Moreover, AURKA-PLK1-FOXM1 axis was over-expressed and hyper-activated in the BCR-ABL1+/CD34+ fraction, which exhibits intrinsic resistance to TK inhibitors [4, 6]. As far as the LSC compartment of CML is concerned, it is worth to remark that the CD34 expression alone is not its phenotypic marker. Other phenotypic features, such as CD25, Lin and CD26 had to be considered as typical traits of such a compartment [29]. However, the CD34+ cells own a sufficient degree of stemness, since they allow bone marrow engraftment in animal recipients, while providing adequate sources for biomolecular analyses [30].

The role of AURKA, PLK1 and FOXM1 in cancer pathogenesis and progression arises from their interaction with β-catenin, which is a central signal for normal and CML stem cells. AURKA sequesters Axin out of the β-catenin destruction complex, PLK1 phosphorylates β-catenin at a central residue for regulated activity in M phase and FOXM1 drives β-catenin nuclear import and transactivation [40, 41]. Accordingly, β-catenin antagonists have been recently advanced to clinical trials to prevent or overcome TK inhibitor resistance in CML LSC. Being restricted to leukemic hematopoiesis, AURKA-PLK1-FOXM1 over-expression could be used as a target to inhibit three signals converging on β-catenin in BCR-ABL1+ hematopoiesis sparing the normal counterpart [41, 42].

AURK inhibitors have been advanced for the treatment of drug-resistant CML, including those driven by T315I BCR-ABL1 mutant, through the inhibition of BCR-ABL1 TK [43]. However, pan-AURK inhibitor clinical use has been impaired by extra-hematopoietic toxicity [44]. More recently, AURKA-selective inhibitors with reduced toxicity have been developed. One, in particular, can block the emergence of mutant cells potentially critical for the development of drug resistance [45]. Here we show the significant reduction of the activating phosphorylation of individual components of AURKA-PLK1-FOXM1 axis both in IM-sensitive and IM-resistant K562 cells in response to AURKA, PLK1 and FOXM1 specific inhibitors (Fig. 1b and c). The downstream event of individual drugs is FOXM1 de-phosphorylation inducing, in turn, β-catenin release from its binding, β-catenin cytoplasmic relocation and degradation (Fig. 2). The inhibition of β-catenin transcriptional activity, as expected, blocks BCR-ABL1+ cell proliferation and survival. Further consideration deserves the role of AURKA, PLK1 and FOXM1 in the persistence of LSC, representing, so far, the major limitation to CML cure In particular, FOXM1 inhibition may revoke its ability of conferring stem-like properties to transformed cells either directly or through AURKA recruitment in the nuclear compartment, hence supporting the advantage of specific inhibitors as complementary drugs to deplete the LSC pool [46–51].

Lastly, the AURKA/PLK1/FOXM1 axis inactivation is associated with a significant increase of GADD45a, a downstream target of AURKA central to CML acceleration towards blast crisis through events involving the dominant negative isoform of p30 C/EBPa, p38 and STAT5 [52]. Notably, GADD45a induction in response to AURKA-PLK1-FOXM1 inhibitors may trigger a negative loop resulting in the inhibition of AURKA kinase activity, reestablish intrinsic and extrinsic stress responses, which restore leukemic cell genomic stability, and promote DNA de-methylation, which drive adaptive gene expression [53–56].

## Conclusion

The over-expression and hyper-activation of AURORA A-PLK1-FOXM1 axis is only partly associated with the BCR-ABL1 TK activity and is enhanced by IM resistance in CML. AURORA A-PLK1-FOXM1 over-expression induce IM resistance by activating multiple pathways implicated in proliferation and survival advantage of leukemic hematopoiesis. AURORA A, PLK1 and FOXM1 have a role in the regulation of β-catenin transcriptional activity. Accordingly, the inhibition of individual components of the axis induces β-catenin inactivation and, in particular, its release from FOXM1, cytoplasmic relocation and degradation. Such mechanisms might revoke the proliferative advantage of drug-resistant LSC, which overcomes TK inhibitor therapy.

## Additional files

Additional file 1: **Figure S1.** Dose-response curves performed to verify K562 IM resistance: significant differences in LD50 of IM–sensitive K562 cells (K562-S, blue curve) as against IM–resistant K562 cells (K562-R, red curve; 0.0293 mM vs. 0.1760 mM, respectively) were observed. (JPG 957 kb)

Additional file 2: **Figure S2.** K562-R response to Thiostrepton, Danusertib and Volasertib, relative to ß-catenin interaction with FOXM1. (TIF 54 kb)

## Abbreviations

APC/C-CDH1: Anaphase promoting complex/cyclosome-cadherin1
AURKA: Aurora kinase A
CCR: Complete cytogenetic response
CDK1: Cyclin-dependent kinase 1
CML: Chronic myeloid leukemia
CMR: Complete molecular response
GADD45: Growth arrest/DNA damage-inducible gene
GSK-3β: Glycogen synthase kinase 3 beta
Hh: Hedgehog
IM: Imatinib
LSC: Leukemic stem cells
MCF: Mononuclear cell fraction
MMR: Major molecular response
PLK1: Polo-like kinase 1
PP1γ: Protein phosphatase 1 gamma
PP2A: Protein phosphatase 2
ROS: Reactive oxygen species
SHP1: SRC homology region 2 domain-containing phosphatase 1
TCF/LEF: T cell factor/lymphoid enhancer-binding factor
TK: Tyrosine kinase
