# Editorial expression of concern: Hyper-activation of Aurora kinase a-pololike kinase 1-FOXM1 axis promotes chronic myeloid leukemia resistance to tyrosine kinase inhibitors

**DOI:** 10.1186/s13046-026-03848-7

**Published:** 2026-10-12

**Authors:** M. Mancini, S. De Santis, C. Monaldi, L. Bavaro, M. Martelli, F. Castagnetti, G. Gugliotta, G. Rosti, M. A. Santucci, G. Martinelli, M. Cavo, S. Soverini

**Affiliations:** 1 Dipartimento di Medicina Specialistica, Diagnostica e Sperimentale – DIMES - Istituto di Ematologia “L. e A. Seràgnoli”, Medical School, University of Bologna, via Massarenti, 9, Bologna, 40138 Italy; 2 Istituto Scientifico Romagnolo per lo Studio e la Cura dei Tumori (IRST) Srl Istituto di Ricovero e Cura a Carattere Scientifico (IRCCS), via Piero Maroncelli 40, Meldola, FC 47014 Italy

**Editorial expression of concern: J Exp Clin Cancer Res 38, 216 (2019)**

**https://doi.org/10.1186/s13046-019-1197-9**; Published: 23 May 2019

The Editor-in-Chief would like to alert readers that concerns were raised regarding highly similar images in this article [1]. Specifically, Fig. 1C and E show similar band appearances in corresponding lanes, particularly for P-AK-A(T288) and P-PLK1(T210).

Although the authors provided raw data upon request, they have been unable to sufficiently address these concerns as the raw image files could not be conclusively verified. Readers are advised to interpret the data listed above with caution.

Mancini, S. De Santis, C. Monaldi, F. Castagnetti, G. Gugliotta, G. Rosti, and S. Soverini disagree with this Editorial Express of Concern. L. Bavaro, M. Martelli, M. A. Santucci, G. Martinelli, and M. Cavo did not respond to correspondence from the Publisher about this Editorial Express of Concern.

## References

[1] Mancini M, De Santis S, Monaldi C, et al. Hyper-activation of Aurora kinase a-polo-like kinase 1-FOXM1 axis promotes chronic myeloid leukemia resistance to tyrosine kinase inhibitors. J Exp Clin Cancer Res. 2019;38:216. 10.1186/s13046-019-1197-9. PMC6533706 31122263

