# Supplementary figures and images for: Hyper-activation of Aurora kinase a-polo-like kinase 1-FOXM1 axis promotes chronic myeloid leukemia resistance to tyrosine kinase inhibitors
Source: J Exp Clin Cancer Res. 2019 May 23;38:216. doi: 10.1186/s13046-019-1197-9 (PMC6533706; doi:10.1186/s13046-019-1197-9)

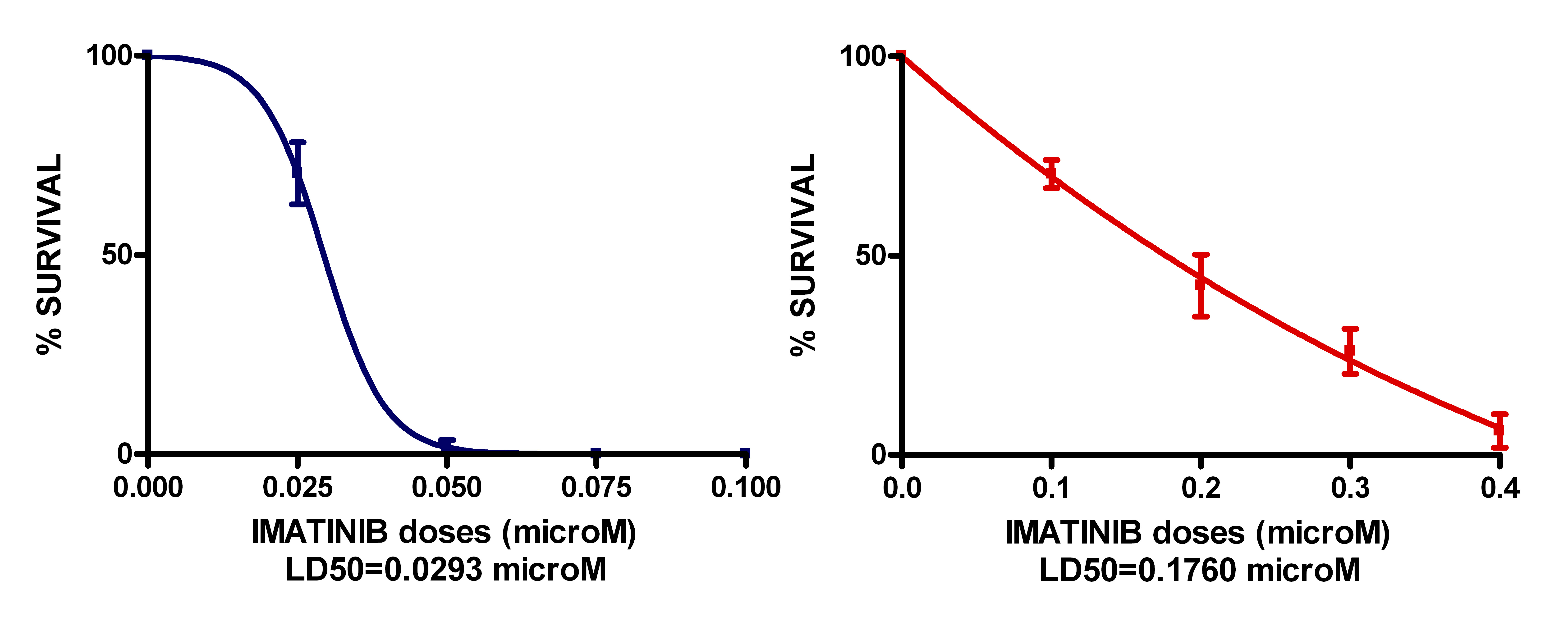

Supplement: Supplementary file 1 — Figure S1. Dose-response curves performed to verify K562 IM resistance: significant differences in LD50 of IM–sensitive K562 cells (K562-S, blue curve) as against IM–resistant K562 cells (K562-R, red curve; 0.0293 mM vs. 0.1760 mM, respectively) were observed. (JPG 957 kb) [file 13046_2019_1197_MOESM1_ESM.jpg]

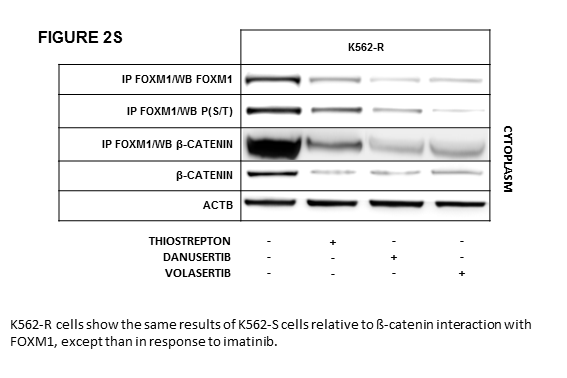

Supplement: Supplementary file 2 — Figure S2. K562-R response to Thiostrepton, Danusertib and Volasertib, relative to ß-catenin interaction with FOXM1. (TIF 54 kb) [file 13046_2019_1197_MOESM2_ESM.tif]
